# The Prevalence and Pattern of Anaemia in Type 2 Diabetics in Ogbomosho, An Urban Community in Southwestern Nigeria

**DOI:** 10.1155/2022/7650015

**Published:** 2022-10-26

**Authors:** Kehinde J. Olufemi-Aworinde, Tolulase A. Olutogun, Joel O. Akande, Roseline O. Akande, Abiona O. Odeyemi, Olufemi J. Idowu, Elizabeth O. Oke, Ademola T. Abolarin, Oluwabukola A. Ala

**Affiliations:** ^1^Department of Haematology and Blood Transfusion, Bowen University, Iwo, Osun, Nigeria; ^2^Department of Chemical Pathology, Bowen University, Iwo, Osun, Nigeria; ^3^Department of Community Medicine, Bowen University, Iwo, Osun, Nigeria; ^4^Department of Medicine, Bowen University, Iwo, Osun State, Nigeria

## Abstract

Anaemia is a frequent finding in type 2 diabetes, but it is typically seen with established chronic kidney disease and renal insufficiency. Cases, where anaemia predates renal insufficiency, are associated with a worse prognosis for the type 2 diabetes patient and an increased susceptibility to complications. This study aims to determine the prevalence and type of anaemia in persons living with type 2 diabetes without established chronic kidney disease in our environment. The study was a hospital-based cross-sectional study that involved 141 people with known type 2 diabetes as the study group and 140 healthy persons as controls. The study population and the controls were selected using a multistage sampling technique. Data were collected using an interviewer-administered semistructured questionnaire at the Endocrinology clinic, Bowen University Teaching Hospital, Ogbomosho. The data obtained were analyzed using the IBM SPSS version 23.0 (*p* value ≤0.05 was considered significant). The biochemical (fasting lipids, HBA1C, FBG, serum albumin, creatinine, urea, uric acid, and insulin) and haematological (FBC and red cell indices; PVC, MCV, MCH, MCHC, and RCDW) parameters of the respondents were analyzed using standard methods. The study showed a statistically significant difference in the prevalence of anaemia among subjects, 69.2% as compared to 30.8% of the control group. Normochromic normocytic anaemia was predominant among the subjects, whereas microcytic hypochromic anaemia was the predominant type in the controls. There was no statistically significant difference between MCV and MCHC of both subjects and controls. There was a positive correlation between the incidence of anaemia and the duration of diabetes among the subjects. More people with type 2 diabetes are now living longer, and the addition of haematological parameters should be part of their baseline investigations to aid in the early detection of complications.

## 1. Introduction

Anaemia is one of the most common preventable public health conditions. Anaemia is defined using the World Health Organization's gender-specific criteria as (Haemoglobin, HB < 13 g/dL in men and <12 g/dL in women) [1]. It is classified into normocytic anaemia when the mean cell volume (MCV) is between 80 and 100 FL, microcytic anaemia when the MCV is less than or equal to 80 FL, and macrocytic anaemia when the MCV is greater than or equal to 100 FL [2].

Diabetes mellitus (DM) is a metabolic disorder with a high prevalence and is associated with disability worldwide. It is defined as persistent hyperglycemia of Glycated haemoglobin A1C, ≥48 mmol/mol (6.5%), fasting plasma glucose level of ≥7.0 mmol/L or random plasma glucose of ≥11.1 mmol/L in the presence of symptoms or signs of diabetes and the presence of relative or absolute insulin deficiency [3]. About 7% of the world's population has diabetes mellitus and 285 million people have type 2 DM worldwide. It is estimated that by the year 2030, the diabetes patient population worldwide will be about 440 million, with its prevalence increasing fast among developing countries [3, 4].

The disease is classified into two predominant types: type 1 DM, characterized by the absence of endogenous insulin, and type 2  DM, marked by insulin resistance [5, 6]. Anaemia resulting from CKD in type 2 diabetes is due to impaired production of erythropoietin by peritubular fibroblasts, nutritional deficiencies (iron, folate, and B12), inadequate response to erythropoietin, and background proinflammatory conditions. However, anaemia can on occasion predate the onset of nephropathy in T2DM [5, 7]. Normocytic normochromic anaemia may be seen in T2DM before the advent of nephropathy. However, the incidence of anaemia without nephropathy is low as documented by previous studies. Anaemia is an independent factor, for the progression to end-stage renal failure among patients with T2DM, thus, having a prognostic implication for diabetics. Anaemia occurs earlier in patients with diabetic renal disease than in nondiabetic individuals with chronic kidney disease [5, 7].

Anaemia in patients with diabetes mellitus has been found to contribute to the pathogenesis and progression of cardiovascular disease and aggravates diabetic nephropathy and retinopathy [8]. It is therefore clearly important to identify anaemia in patients with T2DM before the onset of renal disease.

The prevalence of anaemia in T2DM without established CKD is not well explored in Nigeria. Therefore, this study was carried out to determine the prevalence and type of anaemia in T2DM in the absence of CKD at the Bowen University Teaching Hospital, Ogbomosho.

## 2. Methods and Materials

The study was a hospital-based cross-sectional study done over 12 months at the endocrinology clinic of Bowen University Teaching Hospital in Ogbomosho, Nigeria. The study involved all consenting type 2 diabetes patients aged 15–90 years who met the inclusion criteria. Patient with acutely ill conditions, those with concurrent hemoglobinopathies like sickle cell anaemia and G6PD deficiency, patients with documented or on treatment for nutritional anaemia, and patients who have been diagnosed with chronic kidney disease and renal insufficiency and have commenced management were excluded from the study.

### 2.1. Sample Size Determination and Sampling Technique

The sample size was calculated based on two population mean formulae using G-Power statistical free software version 3.1, by considering the following assumptions: 95% confidence level (2-tailed, *α* = 0.05), 80% power (*β* = 0.20), the ratio of sample size (T2DM/control) was 1 : 1, effect size (*d*) was 0.36 and 10% anticipated nonresponse rate. The sample size was determined to be 141 for the study group and age and sex-matched 140 healthy controls, thus, a total of 281 study participants were included in this study to enhance representativeness. A multistage sampling technique was used.

  Stage 1: The researcher made use of information on patients' cards to sort out those who were being managed for diabetes only at the endocrinology clinic.
  Stage 2: Involved listing eligible patients determined by assessing their serum urea, creatinine, and urinalysis.
  Stage 3: The first eligible respondent was selected by simple random sampling through the balloting method. Then, a systematic random sampling technique was used to select subsequent respondents (*K*th respondent) using the sampling interval obtained from the patient's daily lists throughout the investigation at the endocrinology clinic.

The age and sex-matched healthy controls also had a baseline assessment of their serum electrolytes, urea, creatinine, and glomerular filtration rate (GFR) to rule out underlying renal impairment.

### 2.2. Research Instrument and Data Collection Methods

Sociodemographic and clinical data of the DM and control participants were collected using a semistructured interviewer-administered questionnaire, which was used to obtain important information like age, gender, ethnicity, past and present symptoms of anaemia, past and present treatment of anaemia, and anthropometric measurements, which included height, weight, waist circumference, hip and neck circumference, blood pressure, and body mass index (BMI).

#### 2.2.1. Sample Collection and Preparation

Ten millilitres of venous blood were collected after overnight fasting from the antecubital vein after cleaning with methylated spirit. Each sample collected was separated into three bottles (Two—potassium ethylene diaminotetra acetic, KEDTA, and fluoride oxalate). Whole blood put in bottle 1 was used for full blood count, peripheral blood film, and red cell indices analysis. The second EDTA bottle was separated into plasma by centrifuge and stored frozen for biochemical analysis. Fluoride oxalate was used for fasting plasma glucose.

The laboratory investigations include the full blood count with emphasis on the packed cell volume (PCV) and red cell indices, which are mean corpuscular volume (MCV), mean corpuscular haemoglobin (MCH), mean corpuscular haemoglobin concentration (MCHC), and red cell distribution width (RCDW) using the Mindray haematology autoanalyzer BC-10. Biochemical parameters include fasting lipids (total cholesterol, triglycerides, high-density lipoprotein HDL, low-density lipoprotein, LDL), glycated haemoglobin (HBA1C), fasting blood glucose, serum albumin, creatinine, urea, and uric acid obtained using the JENWAY6305 and UNISPEC semiauto chemistry analyzer. Insulin assay was done using a surgifield SM-MR96A microplate reader. All the parameters were run along with the controls provided by the kit manufacturers.

### 2.3. Measurement of Main Outcome Variables

The following variables were considered: age, sex, weight, height, waist circumference, hip and neck circumference, blood pressure, body mass index (BMI), full blood count including the packed cell volume (PCV) red cell indices, which are mean corpuscular volume (MCV), mean corpuscular haemoglobin (MCH), mean corpuscular haemoglobin concentration (MCHC), and red cell distribution width (RCDW) and patients' diabetes status.

### 2.4. Data Analysis

Data were checked for completeness and consistency and entered into Statistical Package for the Social Sciences (SPSS) version 23 software (IBM Corporation, SPSS Chicago Inc., IL, USA) for analysis. The results were reported as frequency and percentages for categorical variables and mean ± standard deviation (SD) for normally distributed continuous variables. Statistical differences between the groups were determined by the Pearson chi-square test for categorical variables. Differences were considered to be statistically significant at a *p* value less than 0.05.

### 2.5. Ethical Approval and Consent to Participate

Ethical approval was obtained from Bowen University's ethical review committee. Written informed consent was taken from each participant after an adequate explanation of the objectives of the study and the benefits of the study before recruitment into the study. Respondents were told that the participation of persons was voluntary. All other processes of recruitment adhere strictly to the guidelines stipulated by the Helsinki declaration. All information gathered was kept confidential, and participants were identified using only serial numbers.

## 3. Results

A total of two hundred and eighty-one (281) respondents were recruited for the study. One hundred and forty-one (141) were people living with type 2 diabetes (study), while the remaining one hundred and forty (140) were recruited to serve as controls. The two groups were matched for age and sex.

Table 1 shows the sociodemographic characteristics of the respondents. Most of the respondents were from the Yoruba tribe. Only 33.1% of the subjects had tertiary education, compared to 66.7% in the controls. The majority of the participants 114 (67.5%) in the control group were skilled workers, but only 55 (32.5%) were skilled workers in the subject group. The control group had a 113 (56.5%) predominance of married responders, whereas the widowed were more frequent in subject 47 (90.4%). Most of the respondents in both groups were Christians. The average monthly income was lower in the subjects compared with the control group.

Table 2 shows the past and present symptoms of anaemia in both the subject and the control groups. The past symptoms of anaemia (fatigue, pallor, pedal swelling, palpitation, yellow sclera, and passage of coke-coloured urine) were statistically significant in the study group when compared to the control. *p* values of <0.001, 0.008, <0.001, <0.001, 0.014, and 0.004, respectively. The present symptoms of anaemia (fatigue, pallor, and pedal swelling) were also more prevalent in the study group. *p* values <0.01, <0.001 and <0.001 respectively.

Table 3 shows the risk factors for diabetes. The risk factors include a family history of diabetes, smoking, alcohol, physical inactivity, obesity, and stress. The risk factors were more common in the study group than in the controls. *p* values of <0.001, 0.007, 0.003, 0.004, 0.016, and <0.001, respectively.

Table 4 shows the anthropometric measurements and the haematological parameters of the respondents. The mean height of the controls was significantly higher than that of the subjects (1.658 ± 0.084 m vs. 1.604 ± 0.102 m; *p* < 0.001). The mean BMI in the subjects was higher than controls (27.44 ± 5.387 kg/m^2^ vs. 25.98 ± 5.221 kg/m^2^; *p*=0.021). The mean waist circumference was also higher in the subjects than in the controls (95.957 ± 19.343 cm vs. 87.157 ± 18.288 cm; *p* < 0.001). The mean PCV found in subjects (36.565 ± 5.425%) was significantly lower than what was found in the controls (40.751 ± 5.056%) (*p* < 0.001). Similarly, the mean MCH and RCDW of the subjects (26.59 ± 4.784 pg; 15.14 ± 6.435) were significantly lower than what was found in the control group (28.44 ± 6.225 pg; 39.34 ± 6.113) (*p*=0.006, *p* < 0.001), respectively. There was no statistically significant difference in the mean values for MCV and MCHC between the two groups, with the diabetes group having (81.812 ± 23.820 fl; 329.31 ± 53.926 g/l) compared to (82.252 ± 7.579 fl; 334.90 ± 10.669 g/l), respectively, in the control group.

Table 5shows the laboratory parameters of the subjects and the controls. The glycated haemoglobin was statistically significant between the two groups (4.9116 ± 1.4443; 5.4451 ± 0.8170. *p* < 0.001). Moreover, the fasting sugar was significantly elevated in the diabetes group (7.2745 ± 4.24105) compared to the control group (4.8438 ± 0.91974) *p* < 0.001. The insulin assay was higher in the subjects than in the controls (10.4489 ± 11.40753 v 8.0871 ± 10.68929), respectively, but not statistically significant *p*=0.074. The total cholesterol was more in the subjects than in the controls (5.7097 ± 1.63745 v 4.5510 ± 1.43974 mmol/l; *p* < 0.001). The HDL-*c* is lower in the subjects (1.6356 ± 1.0055 v 1.9162 ± 0.79067 mmol/l; *p* < 0.001) while the LDL is higher in subjects (4.7724 ± 11.1792 v 2.4175 ± 1.39066 mmol/l; *p* < 0.001).

**Figure 1:**
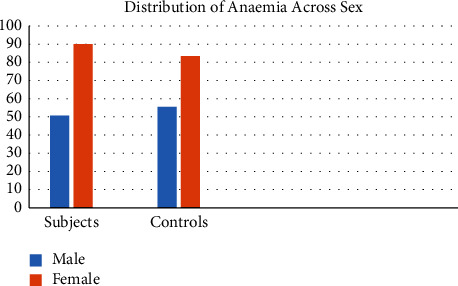
Distribution of anaemia across sex.

Table 6 shows the presence of anaemia in subjects and controls. 69.2% had anaemia in the subjects, whereas only 30.8% had anaemia in the controls. This is statistically significant, with *p* < 0.001. Anaemia is commoner in females in both subjects and controls, as seen in Figure 1.

Table 7 shows the pattern of anaemia in the anaemic subjects, with the majority (66.3%) having normocytic normochromic anaemia.

Table 8 shows the history of DM complications among the anaemic subjects. The complications include retinopathy, diabetic foot, diabetic nephropathy, amputation, diabetic coma, and neuropathy. There was no statistical significance, but complications like retinopathy, diabetic foot, and neuropathy were more common in subjects with anaemia.

## 4. Discussion

The study set out to investigate the presence of anaemia in type 2 diabetics who do not have established chronic renal disease. It also classified any anaemia found using red cell indices as a first step towards understanding the pathophysiology of anaemia in type 2 DM.

This study revealed that diabetes mellitus was more prevalent in people older than forty years old and commoner in females than males, a finding supported by Hillier and Pedula [8]. Most of the participants in the subject group are of the Yoruba tribe, with only a few having no formal education. The majority had at least a primary school education; most were skilled and semiskilled; the majority were married and had an average monthly income of <20.000 nairas; and many of the participants had been diagnosed with type 2 diabetes for more than 5 years. These findings were largely linked to the study area (Ogbomosho), where most inhabitants are of Yoruba ethnic descent, Christians, with varying degrees of skilled and semiskilled workers.

Eighty-three out of one hundred and forty-one subjects (69.3%) had anaemia compared to 37 (30.8%) in controls. This is in keeping with previous studies that anaemia is common in patients with diabetes mellitus [9, 10]. This finding is similar to other studies set in similar socioeconomic and geographical locations as Nigeria. This suggests that the similarities in socioeconomic status and geography may have directly or indirectly affected the cause of anaemia in T2DM [11–14].

In some other studies, anaemia was not a prominent finding amongst type 2 diabetes patients that predated chronic kidney disease [10, 15–17]. The reason for the difference in prevalence may be due to environmental factors such as altitude, humidity, the proliferation of malaria where subclinical infections are common, and the length of time of the disease [5, 18]. It was found that not only was clinical anaemia more common in type 2 diabetes, but the symptoms and the subjective experience of anaemia were more prevalent in type 2 diabetics both past and present. This also corroborates the fact that anaemia and diabetes mellitus might have coexisted before the identification of anaemia [10, 13, 17, 19]. It is also significant to note that a third of the controls also had anaemia. This suggests that anaemia is not only a problem seen in people living with DM but also in the average Nigerian. This may be due to the poor nutritional status found in developing nations like Nigeria [20].

The commonest risk factor for diabetes mellitus in this study is family history. Aravinda in India also found family history and obesity as significant risk factors for the development of diabetes mellitus [14]. However, Barbieri et al. found hypertension and body mass to be significant risk factors. Other significant risk factors were physical inactivity, smoking, alcohol intake, and obesity [13]. All these have been previously documented as significant risk factors by previous studies [14, 19, 21]. The BMI and waist circumference of subjects in this study were statistically significant compared to the control, and this is in agreement with the findings of other authors [19, 21, 22]. These anthropometric parameters are indicators for metabolic syndrome, which is common among people living with diabetes [23, 24].

The packed cell volume, MCH, MCV, and MCHC findings in this study were similar to the findings of Arkew et al. but the RCDW was different [16]. In both studies, anaemia was more prevalent among the type 2 diabetics, but RCDW was much higher in the controls in this study, and significantly increased in their subjects [16]. The reason may be due to different geographical locations and diets. Normochromic normocytic anaemia is the commonest type in this study and it is similar to findings by other authors [13, 25]. This is consistent with anaemia of chronic disease, as found also by Abate et al. [12] pointing out the chronic nature of diabetes mellitus. This is also in consonance with the findings that normochromic normocytic anaemia predates the onset of nephropathy [8, 10]. Microcytic hypochromic anaemia is also seen in about one-third of the subjects with anaemia as found by another author [12] this is also in support of the fact that the aetiology of anaemia is multifactorial [25]. However, megaloblastic anaemia was not detected in either arm of the study. It was discovered that the complications associated with T2DM are more common in those with anaemia. This also supports the fact that quality of life is considered poor in people with T2DM and anaemia [17, 26] and that monitoring and treating anaemia adequately and promptly prevents the development of kidney and cardiovascular diseases [18, 27].

The biochemical parameters in this study showed a statistically significant increase in both glycated haemoglobin and fasting blood sugar in both cohorts in this study. This is in keeping with findings from previous authors [12, 14, 25]. However, this should be within the normal range as found in the controls because the patients are already on one form of treatment or the other. This showed that most patients' blood sugar was not well controlled. Moreover, this is further corroborated by the fact that the insulin assay is higher in subjects than in controls, signifying insulin resistance in T2DM [5]. The total cholesterol and LDL were higher in the subjects than in the controls, while HDL was lower than in the controls. Although the total cholesterol and HDL were statistically significant, LDL and triglycerides were not. This further proves that T2DM had a high prevalence of metabolic syndrome [24].

In conclusion, anaemia in T2DM in this study is high, with anaemia of chronic disease being the most common, and supporting the fact that the anaemia is multifactorial, although the subjects do not have chronic renal disease. This study also supports similar findings that there is insulin resistance in T2DM and that metabolic syndrome is an important component of the disease spectrum. Physicians need to include haematological parameters as part of baseline investigations in T2DM before the onset of complications.

## Figures and Tables

**Table 1:** Sociodemographic characteristics of diabetics and nondiabetics respondents.

Variables	Subjects *n* (%)	Controls *n* (%)
*Age (years)*		
<40	9 (17.0)	44 (83.0)
>40	132 (57.9)	96 (42.1)

*Gender*		
Male	51 (47.7)	56 (52.3)
Female	90 (51.7)	84 (48.3)

*Ethnicity*		
Yoruba	137 (51.5)	129 (48.5)
Igbo	2 (50.0)	2 (50.0)
Hausa	1 (33.3)	2 (66.7)
Others	1 (14.3)	6 (85.7)

*Education level*		
No formal education	37 (92.5)	3 (7.5)
Primary	26 (72.2)	10 (27.8)
Secondary	25 (73.5)	9 (26.5)
Higher institution	43 (33.1)	87 (66.9)
Postgraduate	8 (22.2)	28 (77.8)
Others	2 (40.0)	3 (60.0)

*Occupation*		
Unemployed	2 (14.3)	12 (85.7)
Skilled worker	55 (32.5)	114 (67.5)
Semiskilled	59 (86.8)	9 (13.2)
Unskilled worker	19 (79.2)	5 (20.8)
Retired	6 (100.0)	0 (0)

*Marital status*		
Single	7 (24.1)	22 (75.9)
Married	87 (43.5)	113 (56.5)
Widowed	47 (90.4)	5 (9.6)

*Religion*		
Christianity	122 (49.0)	127 (51.0)
Islam	19 (67.9)	9 (32.1)
Traditional	0 (0)	3 (100.0)
Others	0 (0)	1 (100.0)

*Average monthly income*		
<₦20,000	62 (68.1)	29 (31.9)
₦21,000–₦40,000	24 (53.3)	21 (46.7)
>₦40,000	51 (36.4)	89 (63.6)
Retired	4 (100.0)	0 (0)

*Year diagnosed with the disease*		
<5	68 (48.2)	0
5–10	38 (27.0)	0
>10	35 (24.8)	0

**Table 2:** History of past and present symptoms of anaemia diabetics and nondiabetics.

Variables	Subjects (141)	Control (140)	Df	*p* value
*Past*				
Fatigue			1	<0.001
Yes	38 (88.4)	5 (11.6)		
No	103 (43.3)	135 (56.7)		
Pallor			1	0.008
Yes	12 (91.7)	1 (8.3)		
No	129 (48.1)	139 (51.9)		
Pedal swelling			1	<0.001
Yes	17 (100.0)	0 (0)		
No	124 (47.0)	140 (53.0)		
Heart beat			1	<0.001
Yes	34 (89.5)	4 (10.5)		
No	107 (44.0)	136 (56.0)		
Yellow sclera			1	0.014
Yes	6 (100.0)	0 (0)		
No	135 (49.1)	140 (50.9)		
Cola urine			1	0.004
Yes	8 (100.0)	0 (0)		
No	8 (100.0)	140 (51.3)		

*Present*				
Fatigue			1	<0.001
Yes	18 (90.0)	2 (10.0)		
No	123 (47.1)	138 (52.9)		
Pallor			1	<0.001
Yes	6 (100.0)	0 (0)		
No	135 (49.1)	140 (50.9)		
Pedal swelling			1	<0.001
Yes	14 (100.0)	0 (0)		
No	127 (47.6)	140 (52.4)		
Heart beat			1	0.056
Yes	11 (73.3)	4 (26.7)		
No	130 (48.9)	136 (51.1)		
Yellow sclera				0.062
Yes	4 (100.0)	0 (0)		
No	137 (49.5)	140 (50.5)		
Cola urine			1	0.15
Yes	6 (100.0)	0 (0)		
No	135 (49.1)	140 (50.9)		

**Table 3:** Risk factors for DM.

Variables	Subjects (141)	Controls (140)	df	*p* value
*Family history DM*			1	<0.001
Yes	42 (76.4)	13 (23.6)		
No	99 (43.8)	127 (56.2)		

*Smoking*			1	0.007
Yes	7 (100.0)	0 (0)		
No	134 (48.9)	140 (51.1)		

*Alcohol*			1	0.003
Yes	13 (86.7)	2 (13.7)		
No	128 (48.1)	138 (51.9)		

*Physical inactivity*			1	0.004
Yes	8 (100.0)	0 (0)		
No	133 (48.7)	140 (51.3)		

*Obesity*			1	0.016
Yes	12 (80.0)	3 (20.0)		
No	129 (48.5)	137 (51.5)		

*Stress*			1	<0.001
Yes	32 (80.0)	8 (20.0)		
No	109 (45.2)	132 (54.8)		

*Others*			1	0.502
Yes	1 (100.0)	0 (0)		
No	140 (50.0)	140 (50.0)		

**Table 4:** Anthropometric measurement and red cell indices of diabetics and nondiabetics respondents.

Variables	Categories	Means ± SD	df	*p* value	95% CI	
					Lower	Upper
*Height (m)*	Subject	1.604 ± 0.102	279	<0.001	−0.076	−0.032
	Control	1.658 ± 0.084				

*BMI (Kg/m* ^*2*^)	Subject	27.44 ± 5.387	279	0.021	0.222	2.714
	Control	25.98 ± 5.221				

*BP diastolic (mmHg)*	Subject	81.170 ± 15.527	279	0.672	−2.471	3.826
	Control	80.492 ± 10.865				

*Waist circumference (cm)*	Subject	95.957 ± 19.343	279	<0.001	4.378	13.221
	Control	87.157 ± 18.288				

*Weight (Kg)*	Subject	71.111 ± 13.808	279	0.678	−2.541	3.901
	Control	70.431 ± 13.621				

*Hip circumference (cm)*	Subject	103.91 ± 15.906	279	0.197	−1.458	7.054
	Control	101.12 ± 20.110				

*Neck circumference (cm)*	Subject	37.333 ± 5.520	279	0.074	−0.166	3.532
	Control	35.650 ± 9.684				

*PCV (%)*	Subject	36.565 ± 5.425	279	<0.001	−5.417	−2.954
	Control	40.751 ± 5.056				

*MCV*	Subject	81.812 ± 23.820	279	0.835	−4.597	3.717
	Control	82.252 ± 7.579				

*MCHC*	Subject	329.31 ± 53.926	279	0.231	−14.725	3.563
	Control	334.90 ± 10.669				

*M*	Subject	26.59 ± 4.784	279	0.006	−3.149	−0.543
	Control	28.44 ± 6.225				

*RCDW*	Subject	15.14 ± 6.435	279	<0.001	−25.681	−22.732
	Control	39.34 ± 6.113				

**Table 5:** Biochemical parameters of diabetics and nondiabetics respondents.

Variables	Categories	Mean	SD	df	*p* value	95% CL	
						Lower	Upper
*Total cholesterol (mmol/L)*	Subject	5.7097	1.63747	279	<0.001	0.796	1.520
	Control	4.5510	1.43974				

*Triglyceride (mmol/L)*	Subject	0.5645	0.76538	279	0.158	−0.317	0.052
	Control	0.6974	0.80927				

*HDL-C (mmol/L)*	Subject	1.6356	1.00546	279	0.01	−0.49313	−0.06810
	Control	1.9162	0.79067				

*LDL-C (mmol/L)*	Subject	4.7724	11.17922	279	0.014	0.48079	4.22906
	Control	2.4175	1.39066				

*Glycated haemoglobin*	Subject	4.9116	1.44434	279	<0.001	−0.809	−0.257
	Control	5.4451	0.81705				

*Fasting blood glucose*	Subject	7.2745	4.24105	279	<0.001	1.70902	3.15277
	Control	4.8436	0.91974				

*Serum albumin (mmol/L)*	Subject	46.9641	10.83430	279	0.037	0.12463	4.11932
	Control	44.8421	5.19031				

*Creatinine (mmol/L)*	Subject	62.9433	31.43787	279	0.386	−3.4813	8.9778
	Control	60.1950	20.41335				

*Urea (mmol/L)*	Subject	4.1599	1.97149	279	0.896	−0.43228	0.49357
	Control	4.1293	1.97059				
	Control	134.6143	134.10463				

*Insulin*	Subject	10.4489	11.40753	279	0.074	−0.23475	4.95833
	Control	8.0871	10.68929				

**Table 6:** Presence of anaemia in subjects and controls.

Variables	Subjects	Controls	df	*p* value
Anaemia	83 (69.2)	37 (30.8)	1	<0.01
Normal	58 (36.0)	103 (64.0)		

**Table 7:** The pattern of anaemia among the anaemic subjects (83).

Variables	Frequency	Percentage (%)
Anaemia of chronic disorder/normochromic normocytic anaemia	55	66.3
Iron deficiency anaemia	28	33.7
Megaloblastic anaemia	0	0.0

**Table 8:** History of DM complications among subjects and anaemia.

Variables	Anaemia	Normal	Df	*p* value
*Diabetic retinopathy*			1	0.564
Yes	18 (54.5)	15 (45.5)		
No	65 (60.2)	43 (39.8)		

*Diabetic foot*			1	0.228
Yes	11 (73.3)	4 (26.7)		
No	72 (57.1)	54 (42.9)		

*Diabetic nephropathy*			1	0.383
Yes	2 (40.0)	3 (60.0)		
No	81 (59.9)	55 (40.4)		

*Foot/toe amputation*			1	0.230
Yes	0 (0.0)	1 (100)		
No	83 (59.3)	57 (40.7)		

*Diabetic coma*			1	0.924
Yes	4 (57.1)	3 (42.9)		
No	79 (59.0)	55 (41.0)		

*Diabetic neuropathy*			1	0.798
Yes	26 (60.5)	17 (39.5)		
No	57 (58.2)	41 (41.8)		

*Other complications*			1	0.506
Yes	3 (75.0)	1 (25.0)		
No	80 (58.4)	57 (41.6)		

## Data Availability

The data can be made available from the corresponding author upon request.
